# Besim Ömer: Founder of the first modern maternity hospital and midwifery education in Turkey

**DOI:** 10.18332/ejm/120111

**Published:** 2020-06-01

**Authors:** Asiye Kocatürk

**Affiliations:** 1 Department of Midwifery, School of Health Sciences, Istanbul Medipol University, Istanbul, Turkey

**Keywords:** maternity hospitals, demirkapi viladethanesi, Besim Ömer

## Abstract

The First Maternity Hospital in Turkey was founded in 1892, next to the Medical School in Demirkapi, Istanbul. It was inaugurated by Besim Ömer who went to France in 1887 to study, was very impressed with the practices there and when he returned home, he made great efforts to open the first maternity hospital. Besim Ömer emphasized how vital the first maternity hospital was for pregnant women, newborns, doctors, and midwives. Aiming to minimize maternal and infant mortality and to train informed midwives, these new maternity homes form the basis of today’s maternity clinics.

## COMMENTARY

### Why was the maternity hospital considered as ‘the house of the illegitimate’?

**Figure 1:**
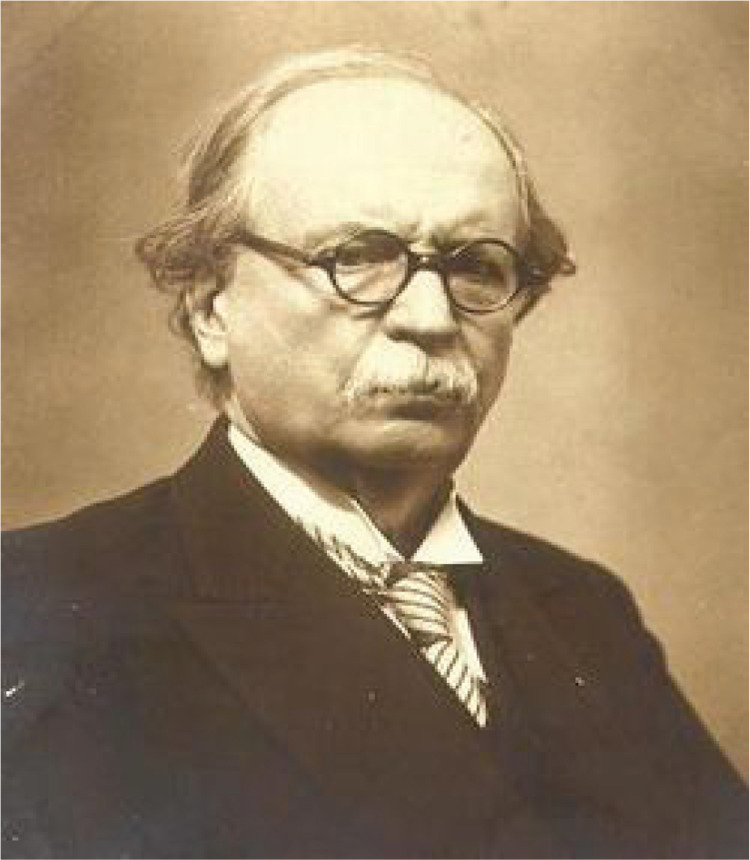
Besim Ömer (1861–1940)^4^

For a very long time in the Ottoman Empire, childbirth was a practice involving the help of midwives at home. In the Ottoman Palace, palace midwives were giving birth in rooms carefully prepared for deliveries, and at the end, they would receive a large sum of money and gifts. People would prepare for a homebirth, with a carefully chosen midwife. Poor families also continued this tradition by using midwives, relatives and neighbours. Those who could not give birth at home were those who had illegitimate children and did not want their parents to know about the child. For this reason, it was thought that only these women would give birth in the hospital. Besim Ömer one of the contemporary doctors (Figure 1), knew that the midwife, who was ready for normal birth, could not know what to do in case of the slightest difficulty, and eventually a physician would be called. As it was usually late to notify the doctor, either the mother or the baby or both often perished. After a normal birth, the dangers for both the mother and the baby persisted, with frequent deaths from illness or lack of care^1,2^.

**Figure 2:**
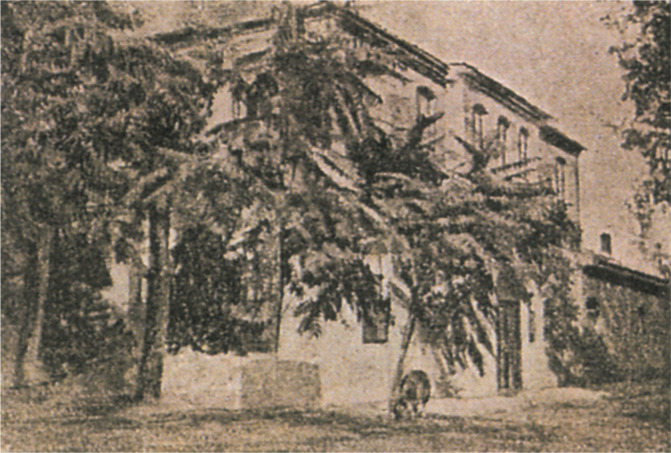
First Viladethane from the book of Besim Ömer^2^

Aiming to minimize maternal and infant mortality and to train informed midwives, the critical part of Ömer’s work was accomplished when a building was ready and equiped (Figure 2). Now it was time to overcome an even more important obstacle. This important stage was to convince the people to give birth in the clinic not at home. Doctors and students were trying to persuade pregnant women to come to the clinic. Besim Ömer wrote articles in newspapers emphasizing with all his efforts the importance and necessity of the maternity hospital^3,4^.

### The maternity hospitals are important for women who give birth

Besim Ömer noted that the clinic is also important for the protection of the health of the mother during and after birth and that the mothers who cannot be provided with care in their homes under adverse conditions can have everything necessary in the maternity hospital:

‘Mothers who need care and happiness and health, their babies who also need special care for any reasons or those who suffer are the ones who this clinic is especially for. They will find the peace and joy of being cared in the clinic. A bed and blanket, a knowledgeable midwife and daily needs including milk and other things are among the supplies the clinic provides. Those are the mothers that will feel the relief. ’^1,3^

### The maternity hospitals are a kind of charity

Besim Ömer writes that each mother and baby who carries risks, like many poor and helpless pregnant women, needs the clinic, so this is a place to be utilised. Moreover, Besim Ömer stated:

‘Although they decided to give birth in their homes in Europe, many pregnant women were immediately transferred to the hospitals due to issues they later faced. In an informal setting, it is not possible to know the kind of intervention and medicine type needed. For that reason, this hospital is not only for the poor and helpless but for the rich as well. The necessity will be appreciated in time.’^3^

### The maternity hospital is a school

Besim Ömer pointed out that the hospital has two kinds of services: one is ‘the service to pregnant women in need’, and the other is ‘a school for nurses and midwives’. In his second year of teaching in military medicine, he was appointed as the principal of the midwifery school in 1895. He spent most of his life training the women who would take on this very important task. He wrote:

‘Most midwives are unable to distinguish between a simple, natural birth and a state of birth that requires medical intervention’, he said. ‘With the facility of such a practical school, better midwives will be trained.’^4^

### The hospital will serve for the Ottoman population and Islam

Besim Ömer said the hospital was also very important to increase the Ottoman population. He stated his belief that these services would be delivered to all parts of the Ottoman Empire with the practical knowledge given to midwives and medical professionals, and that this would serve to increase the Ottoman population. The articles of Besim Ömer in the newspapers gave results, people were convinced of the necessity of the hospital, and this place was made an institution of choice for pregnant women. The hospital founded by Besim Ömer in Demirkapi served for 17 years. Births that needed intervention were delivered to the hands of physicians who were trained on this issue. Both pregnant women and newborns were treated. Babies requiring special care were raised. In the meantime, medical students and midwives were given practical training. The public was educated about it. The problems of the early days were solved between the walls of this hospital. Challenges were overcome with a handful of people. The happiness of the mothers whose lives were saved, the little cries of the babies who embraced life, the footsteps of the doctors, midwives and students who rushed to serve, all echoed in this small building.

**Figure 3:**
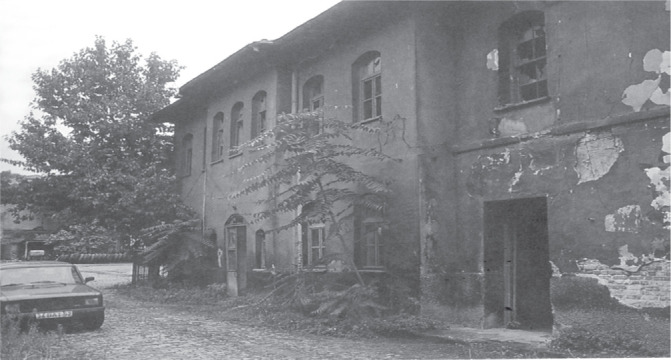
Viladethane building in 19975

**Figure 4:**
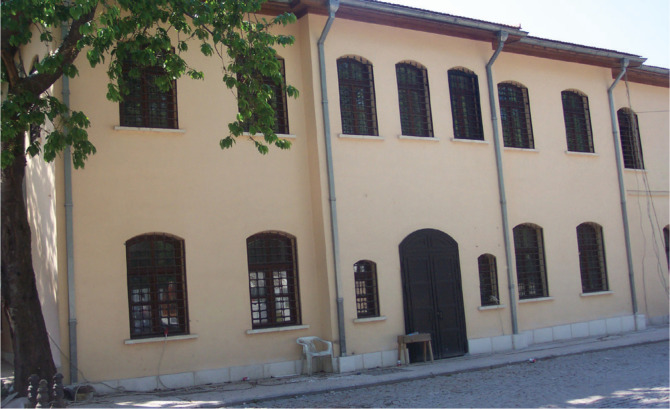
Viladethane restored^5^

This first hospital was in a dilapidated state until the 2000s (Figure 3). Its important task was not considered when it was restored again, as it was merged with the buildings next door. The last version of this building, which has been standing for 125 years, is shown in Figure 4^4,5^.

### Contribution of Besim Ömer to the midwifery profession

In 1839, during the Ottoman period, Tibbiye-i Aliye-i Şahane was opened in Galatasaray. In the French Ordinance of this Medical School, the First Midwifery Training started in 1842, by establishing two courses for birth, specifically for medical students and midwives.

After 1885, Besim Ömer started teaching at the Medical School and Midwifery Course. In 1895, Besim Ömer assumed the training of the Midwifery School, and he was the first midwifery school principal. In 1909, Haydarpaşa Medical Faculty was established by combining civil and military medicine with new staff and the first midwifery school and gynaecology clinic were opened in the buildings in Kadirga, which was emptied of civil medicine. In 1920, Besim Ömer brought elementary school graduate girls from various provinces to this school, in Kadirga, in the Hilal-i Ahmer Barracks where they boarded for 2 years and the need for midwives was met.

## CONCLUSION

The first midwifery school opened by Besim Ömer after the declaration of the Turkish Republic, started to take middle school graduates for training as midwives. The students were obliged to perform compulsory service for 2 years after graduating from their studies for 3 years in the school. In 1924, this school was fully connected to the Faculty of Medicine. Besim Ömer was one of the first pioneers in the development of the nursing and midwifery profession in Turkey^2,3^.
